# Psychometric Assessment and Gender Invariance of the Polish Version of the Gaming Disorder Test

**DOI:** 10.1007/s11469-022-00929-4

**Published:** 2022-10-05

**Authors:** Andrzej Cudo, Christian Montag, Halley M. Pontes

**Affiliations:** 1 Department of Experimental Psychology, The John Paul II Catholic University of Lublin, Lublin, Poland; 2 Department of Molecular Psychology, Institute of Psychology and Education, Ulm University, Ulm, Germany; 3 Department of Organizational Psychology, Birkbeck, University of London, London, UK

**Keywords:** Gaming Disorder, Gaming Disorder Test, Gender measurement invariance, Gamers, Internet Gaming Disorder

## Abstract

In 2019, Gaming Disorder (GD) was officially recognised by the World Health Organization (WHO) as an official mental health disorder. Currently, the Gaming Disorder Test (GDT) is one of the most widely utilised psychometric tools to assess GD symptoms according to the WHO framework. In this context, the present study aimed to develop the Polish version of the GDT so that this tool can be used within the Polish cultural context. Additionally, the research aimed to ascertain the psychometric properties and appropriateness of the Polish GDT. To achieve this goal, the Polish GDT was investigated in terms of its validity, reliability, and gender measurement invariance through two studies. Accordingly, study 1 recruited a total of 675 gamers (340 female; Mean_age_ = 31.74 years; *SD* = 7.75 years; range: 15–45 years) and study 2 comprised 575 gamers (275 female; *M*_age_ = 29.45 years; *SD* = 4.25 years; range: 18–35 years). A Confirmatory Factor Analysis (CFA) was used to assess psychometric properties of the Polish GDT. The reliability was assessed using Cronbach’s alpha, McDonald’s omega, and Average Variance Extracted. Gender measurement invariance was investigated using multiple-group CFA, and the unidimensionality was tested using mean of item residual absolute loadings (MIREAL), explained common variance (ECV), and unidimensional congruence (UC). The results of the psychometric analysis showed that the Polish GDT had a one-factor structure. Additionally, the present study demonstrated that the Polish GDT exhibited adequate levels of convergent validity, reliability, and strict gender measurement invariance. These findings suggest that the Polish version of GDT is a suitable psychometric test to assess GD and facilitate research on GD.

**Supplementary Information:**

The online version contains supplementary material available at 10.1007/s11469-022-00929-4.

Playing electronic games is an increasingly common way of spending leisure time, and the number of active gamers is steadily increasing year by year (Statista, 2021a, b). Currently, the latest reports indicate that there are nearly 227 million gamers of all ages in the United States of America alone (Entertainment Software Association, 2021). Additionally, other recent figures suggest that there are an estimated total of 46.7 million gamers in the United Kingdom, 39.1 million in Germany, 36.5 million in France, 30.6 million in Italy, and 24.6 million in Spain (Statista, 2021c). In Poland, the Polish Games Research ‘20 report (Polish Gamers Observatory, 2020) found that over three quarters of the Polish internet users aged between 15 and 65 years played an electronic game at least once within the recent month. As reported in recent studies, excessive gaming might be an important factor associated with Gaming Disorder (GD) (see also a recent work on time spent gaming and Gaming Disorder symptom load by Pontes et al. (2022)).

In terms of the ongoing debates pertaining to the intersection between mental health and gaming, scientists have investigated both possible positive (see Colder Carras et al., 2018; Griffiths, 2019) and negative (see Kort-Butler, 2021; Przybylski, 2014) consequences associated with gaming. As previously argued by many scholars (e.g. Pontes, 2018), one of the key negative consequences associated with excessive gaming might be the development of addictive behaviour towards the activity (Griffiths et al., 2012; Pontes & Griffiths, 2014), alongside potential aggressive behaviours (Kim et al., 2008), with more recent evidence not fully supporting such relationship (see Verheijen et al., 2021).

A recent review and meta-analysis (Stevens et al., 2021) reported that the worldwide prevalence of disordered gaming was 3.05%, with a confidence interval between 2.38 and 3.91%. Additionally, previous studies reported comorbidities of disordered gaming with other psychiatric disorders such as depression (Ostinelli et al., 2021), attention-deficit hyperactivity disorder (Dullur et al., 2021), obsessive–compulsive disorder (González-Bueso et al., 2018), anxiety disorder (Wang et al., 2017), disorders due to substance use (Burleigh et al., 2019), and other behavioural addictions (Rozgonjuk et al., 2021), such as social media addiction (Pontes, 2017). It should also be noted that more recent studies also reported links between GD and psychological problems in daily life (e.g. Männikkö et al., 2020; Moore et al., 2022).

## Disordered Gaming Frameworks

Taking into account the increasing number of studies reporting the potential addictive effects of video games (Montag et al., 2019a, 2021a; Pontes, 2018; Stevens et al., 2021), before the official GD criteria proposed by the World Health Organization (WHO), the American Psychiatric Association (APA) introduced ‘Internet Gaming Disorder’ (IGD) in the fifth revision of the *Diagnostic and Statistical Manual of Mental Disorders* (DSM-5; APA, 2013) in 2013. Within the DSM-5, IGD was included under Section III as a ‘Condition for Further Study’ as further research on this issue was required as pointed out (APA, 2013). According to the APA framework, IGD may be present, when at least five of the following nine diagnostic criteria occur within the last 12 months: (1) preoccupation with gaming; (2) withdrawal symptoms when gaming is taken away; (3) tolerance; (4) unsuccessful attempts to control participation in games; (5) loss of interests in previous hobbies and entertainment as a result of, and with the exception of, games; (6) continued excessive use of games despite knowledge of psychosocial problems; (7) deception of family members, therapists, or others regarding the amount of gaming; (8) use of games to escape or relieve a negative mood (e.g. feelings or helplessness, guilt, anxiety); and (9) jeopardising or losing a significant relationship, job, or educational or career opportunity because of participation in games (APA, 2013).

Further advances in research lead to the inclusion of GD in the 11th revision of the *International Classification of Diseases* (ICD-11; World Health Organization, 2018) . The WHO framework proposed that GD is characterised by a pattern of persistent or recurrent gaming behaviour manifested by (1) impaired control over gaming, (2) increasing priority given to gaming to the extent that gaming takes precedence over other life interests and daily activities, and (3) continuation or escalation of gaming despite the occurrence of negative consequences (WHO, 2018). Aside from this, the WHO formulated that the gaming activity must result in significant impairment in everyday life, hence the severity level must be given.

It should be noted that contrary to IGD, which was proposed in DSM-5 as a tentative disorder, GD has been fully recognised as an official mental health disorder in the ICD-11 (WHO, 2018) . Consequently, mental health professionals now have a standardised and formalised set of diagnostic criteria to evaluate this addictive disorder. Currently, psychometric tests assessing disordered gaming based on the WHO framework are needed for health professionals to support the healthcare process so that disordered gaming can be effectively diagnosed and evaluated within a sound psychometric assessment framework (Pontes & Griffiths, 2020).

## Methods for Assessing Disordered Gaming Within the APA and WHO Frameworks

Since the publication of the nine IGD criteria in the DSM-5, several psychometric tests based on the APA framework have been developed (see King et al., 2020 for a review). Existing validated psychometric tests assessing IGD according to the APA framework (e.g. The Internet Gaming Disorder Test [IGD-20 Test; Pontes et al., 2014]; Internet Gaming Disorder Scale-Short Form [IGDS9-SF; Pontes & Griffiths, 2015]) contributed to the unification in the assessment of disordered gaming, and thus to the systematic development of research on disordered gaming assessment based on the APA framework.

For example, IGDS9-SF has been translated into 17 languages (Poon et al., 2021), including Polish (Schivinski et al., 2018). Thus, this psychometric test, on the one hand, helped clinicians assess IGD severity in different countries and, on the other hand, made it possible to standardise assessment of disordered gaming across different countries (see Poon et al., 2021). However, despite the official diagnostic criteria for GD proposed within the WHO framework, there is currently a deficiency of psychometric sound tests to assess GD based on the WHO framework (see King et al., 2020; Pontes et al., 2021). Such tools are much needed as recent studies suggest that prevalence rates of GD may differ when considering disordered gaming as defined within the WHO and APA frameworks in the same group of gamers (Montag et al., 2019b, 2021b; Pontes et al., 2022).

The extant evidence reviewing all available psychometric tests for disordered gaming (Karhulahti et al., 2021) found that the Gaming Disorder Test (GDT; Pontes et al., 2021) was the only psychometric test developed to date exhibiting the highest level of validity in the operationalisation of the GD criteria as defined within the WHO framework. This tool is a brief standardised psychometric test including four items reflecting the GD diagnostic criteria based on the WHO framework. Currently, the GDT has been developed in five different language versions (see https://www.halleypontes.com/gdt for further information), with studies on the psychometric properties of these versions reporting adequate psychometric properties. More specifically, the GDT has been developed and adapted to English (Pontes et al., 2021); Chinese (Pontes et al., 2021), German (Montag et al., 2019b, 2021b), Turkish (Evren et al., 2020), and Spanish (Maldonado-Murciano et al., 2021).

## Importance of Gender Measurement Invariance for Disordered Gaming Assessment

Previous research results indicated that GD occurs more often in male than in female gamers (see Stevens et al., 2021; Su et al., 2020 for review). However, it should be noted that female gamers have different patterns and motivations for gaming compared to their male counterpart (Cudo et al., 2022a, b; Kuss et al., 2022; Lopez-Fernandez et al., 2019a, b; McLean & Griffiths, 2019). Furthermore, existing research (Cudo et al., 2022a, b; Stavropoulos et al., 2013, 2019) demonstrated that female gamers tended to spend fewer hours gaming during the week, to use the smartphone more often as a gaming platform, to play action video games less, and to experience lower levels of online flow than male gamers.

Additionally, Cudo et al., (2020a, b, 2022a) showed differences between female and male gamers in the relationship between GD and early maladaptive schemas, self-control dimensions, and impulsivity dimensions. Moreover, Stavropoulos et al. (2019) showed that the relationship between inattention, hyperactivity symptoms, and GD differed across genders between American and Australian gamers with male American gamers displaying higher levels of inattention and hyperactivity symptoms than Australian gamers.

In a similar vein, Andreetta et al. (2020) reported that the relationship between stress and GD differed across genders and cultural orientation levels, such as vertical individualism (a cultural pattern in which an autonomous self is postulated, but individuals see each other as different, and inequality is expected; Singelis et al., 1995). Specifically, male gamers with higher levels of stress and vertical individualism were at greater risk of GD. Conversely, female gamers with a higher level of stress and vertical individualism were found to present a lower risk of GD. Considering the findings on the differences between male and female gamers as well as between these groups and cultural context in GD, it is important to investigate whether the GDT performs well psychometrically, across both genders via measurement invariance testing. It should be noted that the results of gender measurement invariance analysis are necessary for future research to determine if the GDT can be adequately used between male and female gamers so that their symptom-load can be meaningfully compared using the Polish GDT.

## The Present Study

Despite the many existing versions of the GDT and its widespread use in GD research, there is currently no adaptation and validation of the GDT for any country in East and/or Central Europe. Consequently, the present study aimed to develop the Polish version of the GDT. This goal is paramount to helping advance the field as knowledge about the psychometric properties of the GDT in different cultural contexts can contribute, on the one hand, to improving the reliability and comparability of cross-cultural research, and contribute to the broader discussion about GD based on the WHO framework on the other hand.

Developing the Polish GDT will provide a brief and cost-effective screening tool that health professionals can use to assess the GD symptoms in the Polish cultural context. This study will contribute to assessment of the Polish GDT to ascertain whether it can be a valuable screening tool for psychiatrists and clinical psychologists in the Polish context assessing GD based on the WHO framework. Additionally, considering the differences between females and males in both patterns and gaming motives (see Cudo et al., 2022a, b; Kuss et al., 2022; Lopez-Fernandez et al., 2019a, b; McLean & Griffiths, 2019), it is important to test whether the GDT performs well across both genders psychometrically.

To achieve the goals of the present research, two studies were conducted. Study 1 aimed to develop and test the psychometric properties of the Polish GDT and perform an initial validation of this psychometric test while study 2 was aimed at providing further evidence about the validity of the Polish GDT. Specifically, study 1 sought to analyse the reliability, unidimensionality, and gender invariance analysis of the Polish GDT. Further, the goal of this study was to initially validate the Polish GDT using other GD measures and factors related to GD, such as frequency of gaming and motives for gaming. The aim of study 2 was to extend the validation of the Polish GDT to include factors related to anxiety, stress, depression, immersion, and self-control. In this context, it is expected that the frequency of gaming, motives for gaming, anxiety, stress, depression, and immersion will be positively associated with GD as assessed by the Polish GDT, while self-control will be negatively related to GD as assessed by the Polish GDT.

## Study 1

### Methods

#### Participants and Procedures

Study 1 was carried out on a sample of 675 active video game players (340 female gamers) aged between 15 and 45 years (*M*_age_ = 31.74 years; *SD* = 7.75 years) who completed an online survey. It should be noted that the initial sample included 699 participants; however, 24 were excluded as they were careless responders, because they responded in a similar manner across the survey without changing their response. The overall sample characteristics are presented in Table 1. The maximum sampling error (MSE) was 3.77%.

**Table 1:** Characteristics of the study 1 (*N* = 675) and study 2 (*N* = 575) sample

Variables	Category	Study 1		Study 2	
		*N*	Percentage	*N*	Percentage
Gender					
	Female	340	50.37	275	47.83
	Male	335	49.63	300	52.17
Residence					
	Village	147	21.78	141	24.52
	City up to 20,000 inhabitants	88	13.04	71	12.35
	City from 20,000 inhabitants to 100,000 inhabitants	183	27.11	132	22.96
	City above 100,000 inhabitants	257	38.07	231	40.17
Marital status					
	Single	154	22.81	180	31.30
	Non-marriage relationship	206	30.52	179	31.13
	Married	294	43.56	207	36.00
	Widowed	3	0.44	1	0.17
	Divorced	18	2.67	8	1.39
Variables	Category	Study 1		Study 2	
		Mean	SD	Mean	SD
Age		31.74	7.75	29.45	4.25

Participants were recruited online from the Polish research panel Ariadna, and the study was conducted in compliance with the Declaration of Helsinki and approved by the Institute of Psychology’s Ethical Committee at John Paul II Catholic University of Lublin. According to the Ariadna research panel rules, minors can take part in the research only after their parents provide written consent allowing them to participate. The Ariadna research panel is a nationwide survey in which individuals aged 15 and over can participate. Anyone in this age range can register with the research panel. Each panellist is verified to exclude bots that may auto-complete surveys and multiple accounts by one person. It should be noted that no personal information about the participants was collected during the study. Participants received points for completing the survey, which they could use to receive prizes offered by the Ariadna research panel (e.g. books, games, cosmetics, home electronics). The data collection was conducted in January–February 2021; hence, the present work needs to be seen against the background of the COVID-19 pandemic, which is known to be associated with higher levels of disordered gaming (Rozgonjuk et al., 2022). All participants answered the attention check questions correctly. Additionally, data were checked for careless responders. More specifically, the respondents’ response style was checked for use of the same response scale throughout the survey or the individual methods included in the survey.

The present study was part of a larger research project on disordered gaming and game transfer phenomena (see Ortiz de Gortari et al., 2015). Given the clear focus of the present investigation, only the variables needed to assess the psychometric properties of the Polish GDT were utilised and reported. Furthermore, the data set from study 1 is available from: http://hdl.handle.net/20.500.12153/2125.

### Measures

Sociodemographic questions gathered data regarding participants’ age, gender, residence, and marital status.

The Gaming Disorder Test (GDT) (Pontes et al., 2021), which consists of four items rated on a five-point Likert scale (ranging from 1: ‘never’ to 5: ‘very often’), was used to assess symptoms of disordered gaming as per the WHO framework. GDT total scores can range from 4 to 20, with higher scores indicating greater severity of disordered gaming. Taking into account current classification guidelines (see Montag et al., 2019b; Pontes et al., 2021), gamers may be classed as disordered gamers, when they answer all four items of the GDT with 4—often or 5—very often, as such, in this study nine participants (1.33% of the sample 1) fulfilled the WHO criteria for GD.

To develop the Polish GDT, the original GDT items were initially translated from English into Polish by two independent translators. Next, the consolidated Polish version was devised based on these two translations. The consolidated Polish version of the GDT was then back-translated from Polish into English by three independent translators, and the translated items and instruction compatibility were then discussed between the authors of the main project (see Supplementary Table 1).

The Polish version of the IGDS9-SF (Pontes & Griffiths, 2015) was used in the present study (see Schivinski et al., 2018) to assess disordered gaming as per the APA framework. The IGDS9-SF includes a total of nine items that can be responded to on a five-point Likert scale (ranging from 1: ‘never’ to 5: ‘very often’), with higher scores reflecting greater levels of IGD symptoms. The Polish IGDS9-SF utilised in this study presented with adequate reliability (Cronbach’s alpha = .95).

The Polish version of the Motives for Online Gaming Questionnaire (MOGQ) (Demetrovics et al., 2011; Grzegorzewska & Cierpiałkowska, 2018) was also administered to assess motives for gaming within the present sample. This tool consists of 27 items that can be responded to using a five-point Likert response scale (ranging from 1: ‘almost never/never’ to 5: ‘almost always/always’). Additionally, the items form seven subscales corresponding to the following seven gaming motives: (1) escape, (2) coping, (3) fantasy, (4) skill development, (5) recreation, (6) competition, and (7) social. In the current study, the Cronbach’s alphas for each subscale were as follows: (1) .87 for escape, (2) .81 for coping, (3) .89 for fantasy, (4) .90 for skill development, (5) .83 for recreation, (6) .88 for competition, and (7) .88 for social.

The frequency of gaming during the past 12 months was assessed using the Video Game Questionnaire (VGQ; Green et al., 2017). The test provides information on the frequency of use of specific game genres such as first/third-person shooters, action-RPG/adventure, sports/driving, real-time strategy/MOBA, turn-based/non-action role-playing/fantasy, turn-based strategy/life simulation/puzzle, music games, and other. Answers about the number of hours per week spent playing specific game genres were given using the following six-point scale: 1 — *never*, 2 — *less than 1 h*, 3 — *between 1 and 3 h*, 4 — *between 3 and 5 h*, 5 — *between 5 and 10 h*, and 6 — *more than 10 h*.

### Statistical Analysis

Initially, the correlation between GDT items and GDT total scores was calculated using Spearman correlation coefficients for the whole sample, and separately for female and male participants.

Following this, the psychometric properties of the Polish GDT were assessed by means of Confirmatory Factor Analysis (CFA). Considering differences between female and male gamers in the pattern and motivation of gaming (see Cudo et al., 2022a, b; Kuss et al., 2022; Lopez-Fernandez et al., 2019a, b; McLean & Griffiths, 2019), it was important to verify whether the method to assess GD had the same structure in the group of female and male gamers. Consequently, the CFA was carried out separately for females and males. Considering the ordinal nature of item responses and that violation of the multivariate normality assumption was detected using Doornik-Hansen test (Doornik and Hansen, 2008) based on chi-square (*χ*^2^) distribution (female gamers: Doornik and Hansen, 2008; *χ*^2^(*df* = 18) = 1380.21; *p* < .001; male gamers: Doornik and Hansen, 2008; *χ*^2^(*df* = 18) = 773.134; *p* < .001), the CFA was calculated using the weighted least squares mean and variance-adjusted estimator (see Bowen & Masa, 2015; DiStefano & Morgan, 2014). The following fit indices were applied as measures of model fit in the CFA: *χ*^2^, root mean square error of approximation (RMSEA), standardised root means squared residual (SRMR), comparative fit index (CFI), and Tucker-Lewis Index (TLI) (Kline, 2011). An adequate model fit may be present when RMSEA and SRMR are lower than 0.08. Additionally, CFI and TLI values higher than .90 allow the conclusion that a model has acceptable fit to the data (Hu & Bentler, 1999; Kline, 2011).

Next, the gender measurement invariance of the Polish GDT was investigated using multiple-group CFA (Brown, 2006). The gender measurement invariance analysis was carried out to verify whether the structure of the Polish GDT was identical in the female and male gamers. The results of this analysis are necessary for future research to determine the level at which results between female and male gamers can be compared using the Polish GDT.

The RMSEA, SRMR, CFI, and TLI fit statistics were used to assess model fit (Kline, 2011). Considering the presence of a violation of the multivariate normality assumption and ordinal nature of item responses, the weighted least squares mean and variance-adjusted estimator was used (see Bowen & Masa, 2015; Brown, 2006; DiStefano & Morgan, 2014). Based on Brown’s (2006) recommendation, gender measurement invariance was evaluated through the following steps: (1) testing the CFA model separately for female and male; (2) testing the equivalent factor structure of the CFA model and items pattern among female and male gamers (configural invariance); (3) testing the CFA model with factor loadings equality (metric invariance); (4) testing the indicator intercepts equality (scalar invariance); (5) testing the indicator residual variance equality (strict invariance); (6) testing the factor variance equality; (7) testing the factor covariance equality (if applicable, i.e. > 1 latent factor); and (8) testing the latent means equality. It should be noted that steps 1 to 5 are tests of measurement invariance, whereas steps 6 to 8 are tests of population heterogeneity (Brown, 2006).

Furthermore, the test of population heterogeneity allows the comparison of disordered gaming levels, in terms of group-based latent mean between female and male gamers in the context of a measurement model. Additionally, this model is adjusted for measurement error. It should also be pointed out that this comparison between female and male gamers may be conducted only when the configural, metric, and scalar invariances are supported (Brown, 2006). Considering Bowen and Masa (2015), Brown’s (2006), and Rutkowski and Svetina’s (2017) recommendation related to conducting measurement invariance analysis with ordinal data, the criteria for identifying measurement invariance were based on non-significant differences across models assessed by the Satorra scaled chi-square difference test (Satorra, 2000). Additionally, considering that Chen’s (2007) criteria may not be appropriate for measurement invariance using ordinal data in the multiple-group confirmatory factor analysis approach (see Bowen & Masa, 2015; Rutkowski & Svetina, 2017; Svetina & Rutkowski, 2017; Svetina et al., 2020); the criteria proposed by Rutkowski and Svetina (2017) was applied. More precisely, the criteria for identifying measurement invariance between the equal factor loadings model compared with the equal form model were a CFI change smaller than − .004, supplemented by an RMSEA change smaller than 0.050 (see Rutkowski & Svetina, 2017). Additionally, the criteria for identifying invariance between the equal indicator intercepts model and equal factor loadings model as well as between the equal indicator error variance model and equal indicator intercept model were a CFI change smaller than − .004, supplemented by an RMSEA change smaller than 0.010 (see Rutkowski & Svetina, 2017). The last criterion testing non-invariance was used for subsequent comparisons. However, considering Brown’s (2006) recommendation and substantial impact of estimation methods on the RMSEA and CFI (see Shi et al., 2020; Shi and Maydeu-Olivares, 2020), the non-significant differences across models assessed by chi-square test should be prioritised as a more robust way of assessing invariance.

Further psychometric testing included assessing Cronbach’s alpha (Cronbach, 1951), McDonald’s omega (McDonald, 1999), and average variance extracted (AVE; Fornell & Larcker, 1981) to assess the internal consistency of the Polish GDT. Additionally, the unidimensionality of the Polish GDT was tested using different coefficients such as the mean of item residual absolute loadings (MIREAL), explained common variance (ECV), and unidimensional congruence (UC) (Ferrando and Lorenzo-Seva, 2017a). The internal consistency and unidimensionality analyses were performed for the whole sample as well as separately for female and male groups.

Moreover, criterion-related validity was investigated by testing the relationship between the Polish GDT scores and the other relevant psychometric tests used in the study for measuring disordered gaming (i.e. IGDS9-SF scores), frequency of gaming (i.e. VGQ scores), and motives for gaming (MOGQ scores). Taking into account previous research reporting a negative relationship between disordered gaming and age (Montag et al., 2019b), this relationship was tested in the present study. The Spearman correlation coefficient was used to calculate the above relationships, and Guilford and Fruchter’s (1973) guideline was used for correlation coefficient interpretation: slight correlation, correlation coefficient below .20; low correlation, correlation coefficient between .20 and .40; moderate correlation, correlation coefficient between .40 and .70; high correlation, correlation coefficient between .70 and .90; and very high correlation, correlation coefficient above .90.

IBM SPSS version 27 (IBM Corp. Released, 2020) was used to compute descriptive statistics and correlation coefficients, and R version 4.1.0 was used with the lavaan (Rosseel, 2012) and semTools package (Jorgensen et al., 2021) for testing gender measurement invariance and reliability analysis. Additionally, FACTOR 10 (Ferrando & Lorenzo-Seva, 2017b) software was used for unidimensionality analysis.

### Results: Study 1

#### Descriptive Statistics

The descriptive analyses and correlation coefficients for the female and male gamers as well as for the whole sample are presented in Table 2. All correlation coefficients were statistically significant (*p* < .001).

**Table 2:** The descriptive statistic, correlations between items and correlations between items and total scores among whole sample (*N* = 675) as well as female (*N* = 340) and male (*N* = 335) gamers

Variables	Mean	SD	[1]	[2]	[3]	[4]
Female gamers						
[1] Item 1	1.65	0.95				
[2] Item 2	1.66	0.92	0.68			
[3] Item 3	1.63	0.92	0.71	0.71		
[4] Item 4	1.51	0.94	0.70	0.71	0.76	
[5] Total score	6.45	3.32	0.87	0.87	0.88	0.81
Male gamers						
[1] Item 1	1.87	1.02				
[2] Item 2	1.97	1.12	0.79			
[3] Item 3	1.91	1.09	0.78	0.81		
[4] Item 4	1.76	1.05	0.81	0.78	0.78	
[5] Total score	7.50	3.90	0.90	0.93	0.91	0.88
Whole sample						
[1] Item 1	1.76	0.99				
[2] Item 2	1.81	1.03	0.75			
[3] Item 3	1.77	1.02	0.76	0.77		
[4] Item 4	1.63	1.00	0.76	0.76	0.78	
[5] Total score	6.97	3.65	0.89	0.90	0.90	0.85

For all correlation coefficients, *p* ≤ .001

#### Confirmatory Factor Analysis

**Fig. 1:**
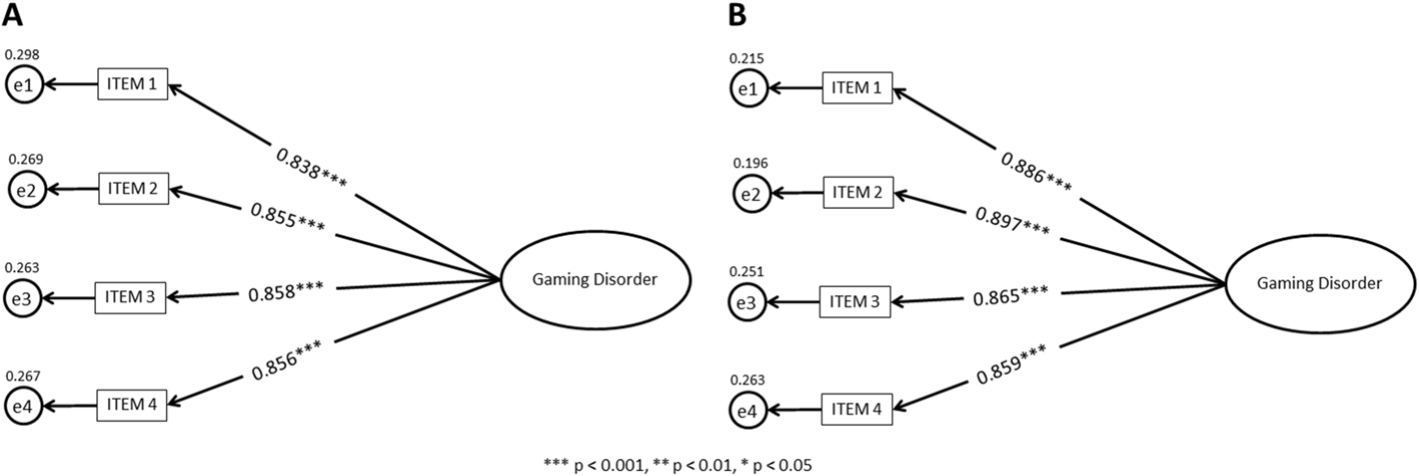
Confirmatory factor analysis results for female (**A**; *N* = 340) and male gamers (**B**; *N* = 335)

The one-factor structure of the Polish GDT fitted well with the data for female gamers: *χ*^2^_(df = 2)_ = 2.41, *p* = .300, RMSEA = 0.025, SRMR = 0.012, CFI = .998, TLI = .995. All standardised factor loadings were statistically significant (*p* < .001), ranging from .84 to .86 (see Fig. 1A). Additionally, for male gamers, the results of the CFA showed excellent fit to the data: *χ*^2^_(df = 2)_ = 4.24, *p* = .120, RMSEA = 0.058, SRMR = 0.013, CFI = .995, TLI = .984, and all standardised factor loadings were statistically significant (*p* < .001), with values ranging from .86 to .90 (see Fig. 1B).

#### Gender Measurement Invariance

In the first step of gender invariance analysis (see Brown, 2006), the CFA model was calculated separately for female and male gamers. The model for female gamers as well as the model for male gamers fitted the data well (see Table 3). Next, the results confirmed equivalent factor structures among female and male gamer groups (i.e. configural invariance; see Table 3): the equal factor loadings model also showed an adequate fit to the data (see Table 3). There was no difference between the configural and metric models. More specifically, the *χ*^2^ test was not statistically significant ( Δ*χ*^2^_(Δdf = 3)_ = 0.56; *p* = .459; see Table 3), and the change in CFI values equalled .004 (ΔCFI = .004). Additionally, the change in RMSEA values was below 0.050 (ΔRMSEA = 0.034).

**Table 3:** Tests of measurement invariance and population heterogeneity of GDT scale among the female and the male gamers group

Model	*χ*^2^	*df*	*p* <	CFI	RMSEA	SRMR	TLI	Model comparison	Δ*χ*^2^	Δdf	*p*	ΔCFI	ΔRMSEA	ΔSRMR	Decision
Models for female and male gamers															
Female model	2.41	2	.300	.998	0.025	0.012	.995	-	-			-	-	-	-
Male model	4.24	2	.120	.995	0.058	0.013	.984	-	-			-	-	-	-
Measurement invariance															
M1: equal form (configural invariance)	6.73	4	.151	.996	0.045	0.011	.987	-	-			-	-	-	-
M2: equal factor loadings (metric invariance)	7.29	7	.400	1.000	0.011	0.018	.999	M1	0.56	3	.459	.004	0.034	0.007	
M3: equal indicator intercepts (scalar invariance)	9.19	10	.514	1.000	0.000	0.019	1.001	M2	1.90	3	.570	.000	0.011	0.001	
M4: equal indicator error variances (strict invariance)	15.57	14	.340	.998	0.018	0.031	.998	M3	6.38	4	.226	.002	0.018	0.012	
Population heterogeneity															
M5: equal factor variance	53.22	15	.001	.941	0.087	0.117	.953	M4	37.65	1	.004	.057	0.069	0.086	
M6: equal latent mean	77.65	15	.001	.904	0.111	0.077	.923	M4	62.08	1	.001	.094	0.093	0.046	

The equal indicator intercepts model (i.e. scalar invariance) showed adequate fit to the data (see Table 3). There were no significant differences between the equal factor loadings model and equal indicator intercepts model: Δ*χ*^2^_(Δdf = 3)_ = 1.90; *p* = .570. Additionally, the change in CFI values was below .002 (ΔCFI = .000), and the change in RMSEA values was almost below 0.010 (ΔRMSEA = 0.011). Consequently, it can be assumed that the indicator intercepts were invariant between the models for male and female gamers.

The equal indicator error variance model displayed satisfactory fit according to the results obtained (see Table 3). There were no differences between the equal indicator intercepts and equal indicator error variance models. More specifically, the *χ*^2^ test was not statistically significant (Δ*χ*^2^_(Δdf = 4)_ = 6.38; *p* = .226; see Table 3). Additionally, the change in CFI values was below .004 (ΔCFI = .002), and the change in RMSEA values was above to 0.010 (ΔRMSEA = 0.018).Consequently, considering the pattern of results, it can be assumed that the indicator error variances were equal between the models for male and female gamers.

Next, the test of population heterogeneity was conducted, with the results of the equal factor variance model presenting no adequate fit according to the fit indices considered (see Table 3). Additionally, there was a difference between the equal indicator error variance and equal factor variance models (Δχ^2^_(Δdf = 1)_ = 37.65; *p* = .004; see Table 3), the change in RMSEA values was above 0.010 (ΔRMSEA = 0.069), and the change in CFI values was above .004 (ΔCFI = .057). Consequently, it can be assumed that there were differences between the male and female gamer groups in terms of factor variance. Taking into account the lack of equality in the factor variance, the factor variance had been unconstrained and the equal latent mean model was additionally compared with the equal indicator error variance model.

More specifically, the equal latent mean model with relaxed factor variance presented lack of fit according to the following fit indices (see Table 3). Additionally, there was a difference between the equal latent mean model with relaxed factor variance and the equal indicator error variance model (Δ*χ*^2^_(Δdf = 1)_ = 62.08; *p* < .001; see Table 3). Additionally, the change in RMSEA values was above 0.010 (ΔRMSEA = 0.093), and the change in CFI values was above .004 (ΔCFI = .094). Consequently, support for population heterogeneity was not found. Furthermore, taking the female gamers group as the reference group, the latent factor mean in the male group was 0.258 (SE = 0.067).

#### Unidimensionality and Reliability Analyses

The MIREAL value was below the recommended threshold of 0.30 for both groups and the whole sample (see Table 4), suggesting that the unidimensional solution of the Polish GDT presented with no substantial bias. The ECV defined as the proportion of common variance attributable to the general factor was above the desired threshold of 0.85 for both groups and the whole sample (see Table 4). Furthermore, the unidimensional congruence was above the recommended threshold of 0.95 for both groups and the whole sample (see Table 4). Taken together, these results suggest that the Polish GDT can be treated as a scale measuring essentially a unidimensional construct.

**Table 4:** Unidimensional and reliability analysis results

		Female gamers	Male gamers	Whole sample
Unidimensionality				
	MIREAL	0.208	0.195	0.129
	ECV	0.928	0.938	0.969
	UC	0.995	0.996	0.999
Reliability				
	Cronbach’s alpha	.914	.929	.924
	McDonald’s omega	.914	.930	.925
	AVE	.726	.769	.754

*MIREAL* mean of item residual absolute loadings, *ECV* explained common variance, *UC* unidimensional congruence, *AVE* average variance extracted

The Cronbach’s alpha of the Polish GDT was above the recommended threshold of .70 (Nunnally and Bernstein, 1994) for both groups and the whole sample (see Table 4). Similarly, McDonald’s omega was above the recommended threshold of .70 for all groups (see Table 4). The AVE was above the recommended threshold of .50 (Fornell and Larcker, 1981) for both groups and the whole sample (see Table 4). Taken together, these results suggest that the Polish GDT presents adequate levels of internal consistency.

#### Criterion-Related Validity

There was a strong relationship between the scores from the GDT and IGDS9-SF (female: rho = .78, *p* < .001; male: *r* = .90, *p* < .001; whole sample: *r* = .85, *p* < .001). Additionally, GDT scores were positively associated with the frequency of different game genres played (see Table 5). The strength of the relationships between GDT scores and frequency of different game genres played ranged from low to moderate. There was a positive relationship between GDT scores and motives for gaming, except for the recreation motive (see Table 5). The strength of the statistically significant relationships between GDT scores and motives for gaming ranged from low to moderate. Last but not least, there was no statistically significant relationship between GDT scores and participants’ age (see Table 5).

**Table 5:** The relationship between IGDS9-SF scale, VGQ subscales, MOGQ subscales, and GDT scale for the female gamers group, the male gamers group and the whole sample

Variables		Female gamers (*N* = 340)	Male gamers (*N* = 335)	Whole sample (*N* = 675)
IGDS9-SF		.78***	.90***	.85***
VGQ				
	First/third-person shooters	.32***	.30***	.34***
	Action-RPG/adventure	.27***	.26***	.29***
	Sports/driving	.33***	.17**	.27***
	Real-time strategy/MOBA	.32***	.34***	.35***
	Turn-based/non-action role-playing/fantasy	.37***	.40***	.40***
	Turn-based strategy/life simulation/puzzle	.17***	.28***	.19***
	Music games	.33***	.43***	.37***
	Other	.30***	.23***	.26***
MOGQ				
	Social	.40***	.44***	.42***
	Escape	.32***	.33***	.32***
	Competition	.31***	.23***	.34***
	Coping	.34***	.37***	.36***
	Skill development	.30***	.21***	.27***
	Fantasy	.33***	.34***	.34***
	Recreation	.05	− .08	− .01
Age		.07	− .03	.06

^***^p < .001; **p < .01; *p < .05

### Study 2

#### Participants and Procedures

Study 2 included a group of 575 active video game players (275 female gamers) aged between 18 and 35 years (*M*_age_ = 29.45 years; *SD* = 4.25 years). The initial sample included 651 participants; however, 76 careless responders were dropped because they responded in a similar manner across the survey without changing their responses and they gave similar answers to the inverted questions as to the direct questions. Similarly to study 1, participants were recruited online from the Polish research panel Ariadna and completed an online survey. The sample characteristics are presented in Table 1. The maximum sampling error (MSE) was 4.09%. The study was conducted in accordance with the Declaration of Helsinki and approved by the Institute of Psychology’s Ethical Committee at John Paul II Catholic University of Lublin. All information about the participants was anonymised and confidential. The data set from the study 2 is available from: http://hdl.handle.net/20.500.12153/2128.

#### Measures

Sociodemographic data was collected by asking participants’ age, gender, residence, marital status.

GDT (Pontes et al., 2021), as presented in study 1, was used to assess levels of disordered gaming according to the WHO framework. In the present study, the Polish GDT presented with adequate reliability (Cronbach’s alpha of .91).

The Polish Depression, Anxiety, and Stress Scale (DASS 21; (Lovibond & Lovibond, 1995; Zawislak et al., 2020) was used to assess negative emotional states such as depression, anxiety, and stress. The DASS-21 includes 21 items belonging to three subscales: depression, anxiety, and stress. Participants responded to the items using a four-point Likert scale (ranging from 0: ‘did not apply to me at all’ to 3: ‘applied to me very much, or most of the time’). Higher scores on each subscale indicate greater levels of depression, anxiety, and stress, respectively. In the current study, the Cronbach’s alphas for each subscale were .93 for depression, .90 for anxiety, and .91 for stress.

The Polish Brief Self-Control Scale (BSCS; Pilarska & Baumeister, 2018; Tangney et al., 2004) was utilised to assess self-control. The BSCS includes 13 items to which answers are given on a five-point Likert scale (ranging from 1: ‘not at all like me’ to 4: ‘very much like me’). The Polish BSCS utilised in this study presented with adequate reliability (Cronbach’s alpha = .83).

The Polish Immersive Experience Questionnaire (IEQ; Jennett et al., 2008; Strojny & Strojny, 2014) was used to assess immersion associated with gaming. The original IEQ contains 31 items; however, four items (i.e. 16, 18, 21, and 22) were removed from the Polish IEQ (Strojny & Strojny, 2014) due to lack of association with the main factor. Participants answered questions that referred to their experience of recently playing their favourite electronic game using a five-point Likert scale (ranging from 1: ‘not at all’ to 5: ‘a lot/very much so’). The Cronbach’s alpha was .89 in the present study.

#### Statistical Analysis

The relationship between depression, anxiety, stress, self-control, immersion levels, and disordered gaming (as assessed by the Polish GDT) was calculated separately for each gender and the whole sample to investigate the criterion-related validity of the Polish GDT. Moreover, Spearman correlation coefficients were calculated for the above relationships, with the Guilford and Fruchter (1973) guideline being used to interpret the coefficients. IBM SPSS version 27 software (IBM Corp. Released, 2020) was used to compute descriptive statistics and correlation coefficients.

### Results: Study 2

#### Criterion-Related Validity

A low and negative correlation between self-control as assessed by BSCS and disordered gaming as assessed by the Polish GDT was observed (see Table 6). Additionally, GDT scores were positively associated with scores of depression, anxiety, and stress as measured by DASS-21. The strength of the relationships between GDT scores and the DASS-21 subscale total scores ranged from low to moderate. There was a moderate relationship between disordered gaming as assessed by the Polish GDT and immersion as assessed by IEQ (female: rho = .55, *p* < .001; male: rho = .50, *p* < .001; whole sample: rho = .52, *p* < .001) (see Table 6).

**Table 6:** The relationship between depression, anxiety, stress as assessed by DAS-12, self-control (BSCS), immersion (IEQ), and Gaming Disorder as assessed by GDT for the female gamers group, the male gamers group, and the whole sample

Variables		Female gamers (*N* = 275)	Male gamers (*N* = 300)	Whole sample (*N* = 575)
BSCS		− .32***	− .32***	− .32***
DASS-21				
	Depression	.36***	.37***	.36***
	Anxiety	.29***	.41***	.35***
	Stress	.31***	.44***	.37***
IEQ		.55***	.50***	.52***

^***^p < .001; **p < .01; *p < .05

## Discussion

The purpose of the present investigation was to develop the Polish GDT and to report its psychometric properties. The results of the CFA and unidimensionality analysis showed that the Polish GDT had a one-factor structure analogous to the original GDT (Pontes et al., 2021). The unidimensional structure of the GDT was also reported across other studies from different cultural contexts (Evren et al., 2020; Maldonado-Murciano et al., 2021; Montag et al., 2019b). In terms of the gender measurement invariance analysis, the findings showed that the Polish GDT presented with strict measurement invariance. More specifically, the same one-factor structure, the same patterns of factor loadings, and equivalent strength of the relationship between the items and main factor were presented for both female and male gamer groups. Additionally, there were equal indicator intercepts and indicator error variances between the female and male gamer groups (see Brown, 2006).

### Gender Invariance Results

These findings were in line with the findings of a previous study (Maldonado-Murciano et al., 2021), supporting strict gender invariance for the Spanish GDT. Similarly to the findings reported in the Spanish GDT study (Maldonado-Murciano et al., 2021), the Polish GDT presented with strict identical structures for females and males. Strict invariance is achieved when the factor loadings, indicator intercepts, and indicator error variances are equal across groups (see Brown, 2006). Consequently, strict invariance guarantees comparability of the GDT across female and male groups. However, it should be noted that support for population heterogeneity was not found in the present study. The factor variance and latent mean were not equal across female and male gamers. Specifically, female and male gamers may differ in terms of disordered gaming symptoms at the latent trait level when the measurement model is adjusted for measurement error. Therefore, male gamers may present higher variance and higher level of disordered gaming symptoms at the latent trait level.

Previous research (Maldonado-Murciano et al., 2021) reported no gender differences between female and male gamers in GDT scores. However, in contrast to the presented studies analysing differences between female and male gamers in latent GDT scores, they used the Mann–Whitney *U* test to compare the GDT total score between males and females. It should be noted that the Mann–Whitney *U* test uses the rank of each case, while the latent score is a factor value calculated from the weighted item score. Additionally, Montag et al. (2019b) reported that the ratio of gamers afflicted with GD did not differ across genders in relation to the WHO framework.

However, they found a low negative relationship between gender and GDT scores in the structural mediation model when the GDT score was treated as a continuous variable. Furthermore, Pontes et al. (2021) reported no relationship between gender and GDT scores in a multiple-indicator multiple-cause model (MIMIC model). However, they found a low negative relationship between gender and GDT scores when the structural model also included loneliness and depression. Taken together, the results of the previous studies using the GDT do not provide conclusive findings on the possible differences between female and male gamers about GD based on the WHO framework. One possible explanation could be related to the differences in GDT score variation between both groups and the weak relationship between GDT scores and gender. Further research is needed to explain the differences between female and male gamers in disordered gaming as assessed by the GDT.

The unidimensionality analysis using different coefficients such as MIREAL, ECV, and UC showed that the Polish GDT had essentially a unidimensional structure. Additionally, the internal consistency of the Polish GDT as assessed with Cronbach’s alpha, McDonald’s omega (McDonald, 1999), and AVE (Fornell & Larcker, 1981) was adequate for the female and male gamers as well as for the whole sample, similarly to previous research reporting robust internal consistency of GDT in English (Pontes et al., 2021), Chinese (Pontes et al., 2021), German (Montag et al., 2019b), Spanish (Maldonado-Murciano et al., 2021), and Turkish (Evren et al., 2020) populations.

### Criterion-Related Validity Results

The criterion-related validity results from study 1 showed a strong relationship between GDT and IGDS9-SF scores. Previous studies (Evren et al., 2020; Maldonado-Murciano et al., 2021; Pontes et al., 2021) also reported strong associations between these two screening tools (Evren et al., 2020; Maldonado-Murciano et al., 2021; Pontes et al., 2021). Additionally, there was a positive relationship between GDT scores and time spent playing video games (i.e. weekly time spent gaming) in the last 12 months (see also Pontes et al., 2022). This relationship was significant for every game genre analysed (i.e. first/third-person shooters, action-RPG/adventure, sports/driving, real-time strategy/MOBA, turn-based/non-action role-playing/fantasy, turn-based strategy/life simulation/puzzle, music games). These results are in line with previous studies on the relationship between time spent gaming and GD (see Cudo et al., 2018, 2020c; Evren et al., 2020; Mihara & Higuchi, 2017; Pontes et al., 2021). It is worth noting that GD is characterised by impaired control over gaming and increased priority given to gaming to the extent that gaming takes precedence over other life interests and daily activities (WHO, 2018). Consequently, greater GD symptom severity may be associated with longer gaming time as well, although this conclusion requires further testing.

The results from study 1 also showed a positive relationship between GDT scores and almost all gaming motives as assessed by the MOGQ, except the recreation motive. Based on single correlation coefficient results, Montag et al. (2019b) reported that GD assessed by GDT was positively associated with all motives assessed with the MOGQ. However, in the mediation model, they showed that GD was positively associated with escape and competition motives, and negatively associated with skill development and recreation motives. Furthermore, Laconi et al. (2017) reported single positive correlations between IGD and all motives for gaming assessed with the MOGQ. However, in the regression model, only escape, competition, coping, and skill development (with negative sign) motives were associated with disordered gaming (see Laconi et al., 2017). In contrast to the previous study (Montag et al., 2019b), in the current study, there was no relationship between GDT scores and age.

For study 2, the criterion-related validity results showed a negative relationship between GDT scores and self-control. These results are consistent with previous research on the relationship between low self-control and GD (see Cudo et al., 2020a; Mills & Allen, 2020; Pontes & Macur, 2021). There was a positive relationship between depression, anxiety, and stress and GD. Maldonado-Murciano et al. (2021) showed that GDT scores were positively associated with DASS-21 subscale scores such as depression and stress. However, they did not find a relationship between anxiety and GD. Maldonado-Murciano et al. (2021) postulated that the lack of a correlation between anxiety and GD might be related to the effects of the COVID-19 pandemic on mental health. Another possible explanation for the differences between the previous study (Maldonado-Murciano et al., 2021) and the current study in the relationship between anxiety and GD may be associated with the cultural differences that were shown so far in the relationship between depression and GD (see O’Farrell et al., 2022).

It should be noted that previous research consistently showed a positive correlation between depression, stress, and anxiety with IGD (see Pontes & Griffiths, 2016; Wong et al., 2020), further highlighting that the nature of the online experience is paramount to explaining detrimental outcomes (Pontes et al., 2015). Additionally, according to the I-PACE model (Brand et al., 2016, 2019), low self-control, depression, anxiety, and stress are key predictors in the development of behavioural addictions such as GD. Moreover, the criterion-related validity results also showed a strong positive correlation between GDT scores and IEQ scores. These results are aligned with previous research (Li et al., 2021), suggesting a link between GD and game flow experience. Additionally, these findings may suggest that GD was positively associated with lack of awareness of time, loss of awareness of the real world, and engagement and sense of being in the game environment during gaming.

### Limitations

The present findings should be interpreted in light of several limitations. Firstly, the current study was a self-report and cross-sectional study. Hence, the study results cannot be interpreted as causal relationships. Secondly, potential careless responding to the survey (see Brühlmann et al., 2020) should be noted. Specifically, despite the use of attention checks in the survey, participants recruited online from the Polish research panel Ariadna may have responded in a biased manner. Thirdly, the research panel may not reflect the population of all gamers, so it is important to be cautious when generalising the results obtained. Fourthly, it should be pointed out that survey studies may have limitations associated with methodological biases (e.g. lack of depth, biased recalls, social desirability). Fifthly, in the present study, no clinical sample was recruited. Consequently, the sensitivity and specificity in the diagnostic accuracy of the Polish GDT were not verified. Thus, further research is needed to ascertain the diagnostic accuracy of the Polish GDT in terms of estimation of cutoff points for the GDT. Sixthly, taking into account the age range of the participants (from 15 to 45 years), one should be cautious about generalising the results to children and older gamers groups (see a recent work by Wernicke & Montag, 2021, 2022 applying the GDT in children). Finally, although the present study results in the Polish context are consistent with findings in other cultural contexts (Evren et al., 2020; Maldonado-Murciano et al., 2021; Montag et al., 2019b; Pontes et al., 2021), caution should be exercised in generalising the results to further cultural context (see Stevens et al., 2021).

## Conclusion

The present study demonstrated that the Polish GDT is a valid and reliable psychometric test for assessing GD based on the WHO framework (WHO, 2018). The Polish GDT presents with a single-factor solution with robust factor loadings. Additionally, findings obtained indicated that the Polish GDT showed strict gender measurement invariance, signifying that the test can be successfully applied as a screening tool to assess GD symptoms among female and male gamers. Moreover, the criterion-related validity results also showed that the Polish GDT was positively associated with other disordered gaming measures and other GD-related variables. Consequently, the Polish GDT can be a valuable screening tool for psychiatrists and clinical psychologists in the Polish context assessing GD based on the WHO framework.

## Supplementary Information

Below is the link to the electronic supplementary material.

Supplementary file1 (DOCX 15 KB)

## Data Availability

The dataset from the present study is available from the institutional repository database (access link: http://hdl.handle.net/20.500.12153/2125 and http://hdl.handle.net/20.500.12153/2128).
