# Supplementary material for: Psychometric Assessment and Gender Invariance of the Polish Version of the Gaming Disorder Test
Source: Int J Ment Health Addict. 2022 Oct 5:1–24. Online ahead of print. doi: 10.1007/s11469-022-00929-4 (PMC9533968; doi:10.1007/s11469-022-00929-4)
Supplement: Supplementary file 1 — Supplementary file1 (DOCX 15 KB) [file 11469_2022_929_MOESM1_ESM.docx]

Supplementary material

Table 1. Items content.

| 1. | Miałam/em trudności z kontrolowaniem mojej aktywności związanej z grami.  [I have had difficulties controlling my gaming activity.] |
| --- | --- |
| 2. | Nadawałam/em coraz większy priorytet graniu nad innymi zainteresowaniami życiowymi i codziennymi czynnościami.  [I have given increasing priority to gaming over other life interests and daily activities.] |
| 3. | Kontynuowałam/em granie mimo pojawienia się negatywnych konsekwencji.  [I have continued gaming despite the occurrence of negative consequences.] |
| 4. | Doświadczyłam/em poważnych problemów życiowych (np. osobistych, rodzinnych, społecznych, edukacyjnych, zawodowych) z powodu nasilenia moich zachowań związanych z graniem.  [I have experienced significant problems in life (e.g., personal, family, social, education, occupational) due to the severity of my gaming behavior.] |
